# The Effectiveness of Machine Learning Algorithms in Predicting Healthcare Service Quality Metrics: A Systematic Review

**DOI:** 10.3390/healthcare14182887

**Published:** 2026-09-08

**Authors:** George Katharakis, Nikolaos Rikos, Michael Rovithis, Dimitrios Papageorgiou, Areti Stavropoulou

**Affiliations:** 1 Department of Nursing, School of Health, Faculty of Health and Care Sciences, University of West Attica, 12243 Egaleo, Greece; dpapa@uniwa.gr (D.P.); astavropoulou@uniwa.gr (A.S.); 2 Department of Nursing, School of Health Sciences, Hellenic Mediterranean University, 71410 Heraklion, Greece; rikosn@hmu.gr; 3 Department of Business Administration & Tourism, School of Management and Economics Sciences, Hellenic Mediterranean University, 71410 Heraklion, Greece; rovithis@hmu.gr

**Keywords:** machine learning algorithms, healthcare service quality, prediction, patient satisfaction, efficiency, systematic review

## Abstract

**Background/Objectives:** Healthcare service quality is a critical dimension of patient safety and operational performance. Traditional assessment approaches are often limited in their ability to support prediction, which has increased interest in machine learning (ML) models. This systematic review assessed the effectiveness of ML algorithms in predicting healthcare service quality metrics, with emphasis on their applications, comparative performance and implementation challenges. **Methods:** A systematic literature search was conducted in PubMed/MEDLINE, Scopus, CINAHL, and ScienceDirect for studies published between 1 January 2020 and 30 July 2025. Following title/abstract screening and full-text review, 49 studies were included. Studies were grouped into conventional/ensemble ML and deep learning categories based on the primary model class analyzed in each article. **Results:** The reviewed studies focused mainly on acute clinical and operational outcomes, especially length of stay (27.0%), mortality (23.0%), and readmission rates (18.0%), while subjective, patient-centered metrics received less attention. Conventional and ensemble ML models, particularly RF and XGBoost, were frequently reported, while deep learning models were used in more complex prediction tasks. **Conclusions:** The evidence suggests that well-validated and interpretable ML models can support healthcare quality prediction. However, important challenges remain regarding implementation, validation, generalizability, and data heterogeneity.

## 1. Introduction

In the last few decades, digital transformation in healthcare has created a fast-changing competitive environment, where healthcare service providers are motivated to embrace patient-centered and high-quality services for patients, both with the help of opportunities and challenges [1]. The complexity of the healthcare sector has increased, and traditional ways of assessing and improving service quality, mostly based on manual audits, surveys, and retrospective analyses, have found it difficult to catch up with the quick changes in the needs of stakeholders and patients [2,3]. In this particular scenario, machine learning (ML) algorithms have emerged as a new and revolutionary approach capable of processing huge datasets to reveal correlations, predict outcomes, and make evidence-based decisions for healthcare distribution [2,3,4,5].

Healthcare service quality is multidimensional by nature, including clinical effectiveness, safety, patient experience, accessibility, and operational competence. Understanding the fact that distributed or too extensive quality assessment instruments are of less help, researchers have focused their efforts on creating a framework of tools, such as CIRMQUAL, which integrates 14 key factors, including quality of care, safety, staff attitude, communication, cost, and waiting time, into four main categories, those being clinical, infrastructural, relationship, and managerial quality factors [2]. Such a designed strategy not only provides a detailed service quality measurement but also reveals suggestions for identified enhancements [2].

Healthcare service quality is treated in this review as a multidimensional construct including clinical effectiveness, safety, patient experience, accessibility, and operational performance [2,3,4]. For consistency, the reviewed outcomes are grouped into clinical outcomes, process and operational indicators, resource-use measures, and patient-reported dimensions, reflecting the broader view that healthcare quality extends beyond mortality, length of stay, and readmission alone [3,4]. The use of machine learning (ML) algorithms such as random forests (RFs), neural networks (NNs), and support vector machines (SVMs) for quality assessment in healthcare is a big step forward for advancements in this sector. These models can predict key service quality metrics, such as patient satisfaction, waiting times, and treatment outcomes, and frequently demonstrate higher predictive accuracy and discrimination compared with traditional statistical techniques [6,7,8]. As an illustration, ML-oriented processes are the reason for the ability to have up-to-date information on patients’ waiting times, which consequently leads to a proactive approach to resource management that results in better patient satisfaction [6,7]. In the case of home-based healthcare, ML models have been useful in predicting service times and streamlining the care delivery process for those patients who have extensive care [9]. Apart from this, ML-driven feature selection and opinion mining have essentially pinpointed the most effective factors that could influence patients’ satisfaction and thereby have assisted in the implementation of more planned and effective quality improvement activities [10,11].

But despite these improvements, the initiation of ML in the healthcare service quality prediction field is not without challenges. Problems such as the quality of data, model explainability or interpretability, and prediction uncertainty are still major issues, particularly in high-risk settings where mistakes may have serious effects [12,13]. Making sure that ML models are fair and not biased is also a critical point in preventing the worsening of existing health inequalities [14]. Furthermore, ML procedures must be accurately placed according to clinical workflows and undergo extensive validation/monitoring to ensure the sustainability of solutions [4,15]. Also, trust development among stakeholders is a prerequisite for success in the implementation of ML solutions [4,15,16,17].

In short, ML algorithms’ practical application in the prediction of healthcare service quality metrics is like a new arrow in healthcare management’s quiver, enabling it to gain momentum [18]. ML can easily support health organizations to prosper by supporting real-time recommendations for regular monitoring, evaluation, and improvement of healthcare, which, on the whole, results in better patient outcomes and greater operational efficiency [19,20]. The ascendant field will not only be sustained by but is also dependent upon the continuing collaboration of researchers, data scientists, clinicians, and healthcare managers to set the foundation of the transformational revolution in healthcare service quality through ML [19,20].

To address the identified gaps in the current literature, the main goal of this systematic research is to find out the usefulness of machine learning algorithms in estimating healthcare service quality metrics by solving three core research questions:

Q1: Which are the most prevalent healthcare service quality metrics that machine learning models target?

Q2: How do various machine learning algorithms compare regarding their effectiveness in predicting healthcare service quality metrics?

Q3: What are the practical implications and limitations of machine learning in predicting healthcare service quality, with a major focus on interpretability, key features, and real-world applications?

Through the detailed assessment of the above research questions, this study will try to present all the required parameters for which ML models could be helpful in providing better healthcare services.

## 2. Materials and Methods

### 2.1. Design

The current research employed a systematic review methodology to thoroughly analyze and synthesize the existing literature on ML applications in the prediction of healthcare service quality. The most recent methodological literature emphasizes the prominence of structured, precise systematic review methods [21,22] and multi-method analytical frameworks [23], particularly in addressing problems of bias and generalizability [24,25].

In accordance with methodological transparency and PRISMA guidelines (Supplementary Materials), the systematic review protocol was prospectively registered with the International Prospective Register of Systematic Reviews (PROSPERO; registration number: CRD420261465111) and is publicly accessible at https://www.crd.york.ac.uk/PROSPERO/view/CRD420261465111 (accessed on 30 July 2026). The publicly accessible record includes the review question, eligibility criteria, information sources, search approach, study selection process, data extraction plan and synthesis strategy.

An extensive literature search across the electronic databases of PubMed/MEDLINE, Scopus, CINAHL and ScienceDirect for articles published between 1 January 2020 and 30 July 2025 was conducted. ScienceDirect was included as a complementary search source to capture in-press, full-text health informatics and multidisciplinary healthcare operations research from major Elsevier journals prior to final indexing in Scopus or MEDLINE. The search strings utilized a combination of controlled vocabulary (MeSH headings in PubMed and CINAHL subject headings) and title/abstract text words with Boolean operators, field restrictions, and truncation symbols.

Table 1 illustrates the conceptual search framework, while the complete database-specific executable search strings, limits, and search dates are reported in Table A1 (Appendix A).

### 2.2. Identifying Relevant Studies and Study Selection

**Figure 1:**
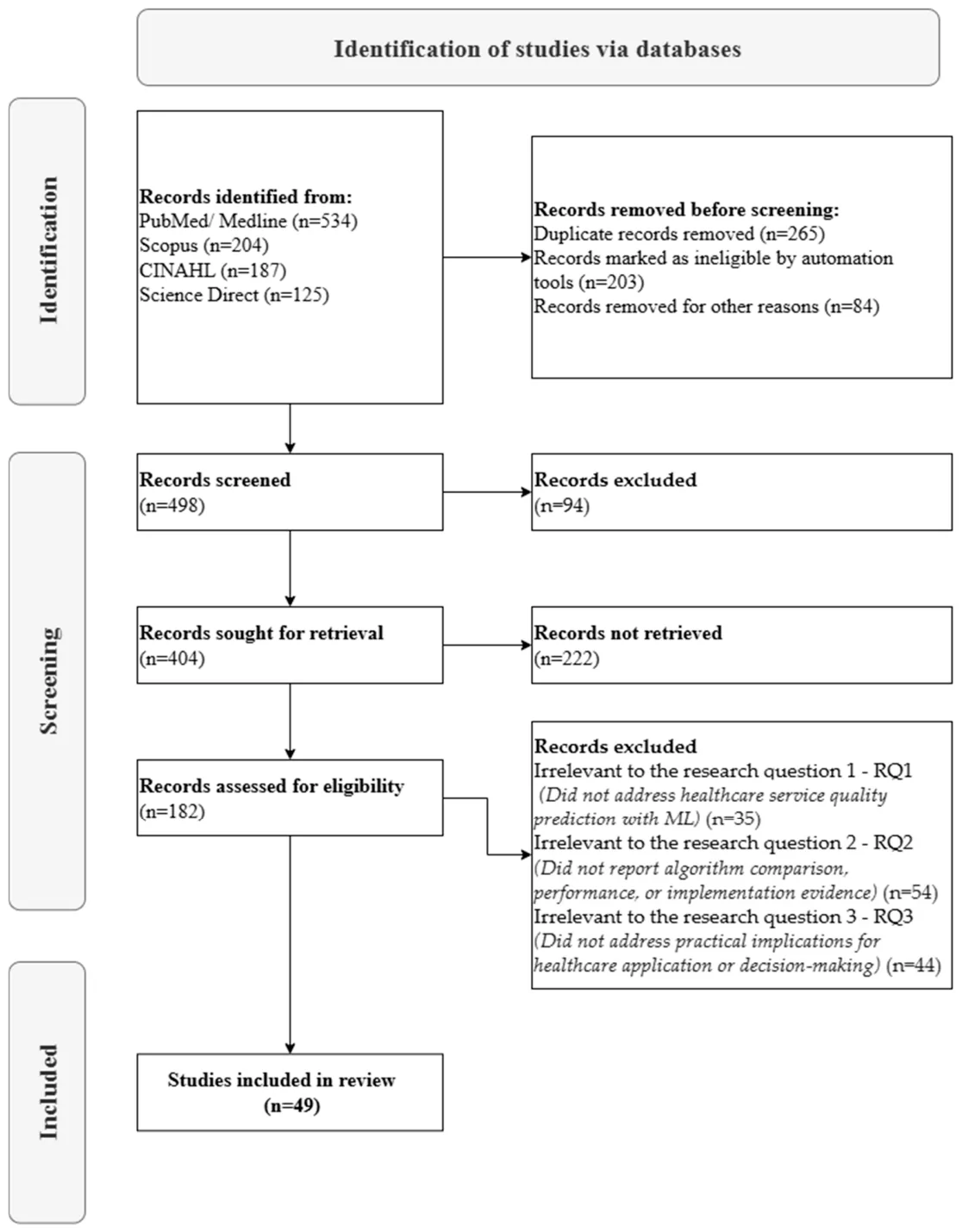
PRISMA flow diagram of the systematic search and selection process.

The systematic review inclusion criteria required original research articles published in English between 2020 and 2025, and employing qualitative, quantitative, or multi-algorithm approaches. Additionally, the articles should mention metrics, algorithm comparisons, healthcare quality prediction with machine learning, and their practical implications as the main topics of the studies. The authors of this research followed a comprehensive multi-tiered process for sifting as per the PRISMA guidelines, which are shown in Figure 1 [21].

This structured method confirmed the complete identification and appraisal of all the evidence, per the pre-established criteria. A detailed search and examination of the databases led to an initial collection of 1050 records. The initial search of the literature, managed using EndNote version 21 (Clarivate Analytics, Philadelphia, PA, USA), was classified and deduplicated through a systematic process, with all automatically identified pairs undergoing manual visual verification by one reviewer prior to deletion to prevent erroneous removal of distinct articles with shared phrasing [26]. After duplicates were removed (*n* = 265), as well as the records flagged as ineligible by automation tools (*n* = 203) and those excluded for other reasons (*n* = 84), a total of 498 studies were subjected to title and abstract screening.

During the title- and abstract-screening process, 94 records were excluded, leaving 404 records sought for full-text retrieval. Of these, 222 records (55.0%) could not be retrieved despite attempts to obtain access through institutional library services, interlibrary loan requests, and direct author contact. The main reasons for non-retrieval included lack of institutional subscriptions, paywalled articles without accessible alternatives, and records unavailable in full-text form. Abstracts alone were not considered sufficient for full eligibility assessment because detailed information on study methodology, model performance metrics, algorithm comparison, validation approaches, and implementation-related findings could not be reliably extracted from them. This high non-retrieval rate represents a potential source of availability bias, and its implications are addressed in the Section 5.

Original research studies employing quantitative, qualitative, or multi-algorithm approaches were eligible. However, their contribution depended on the specific research question. Studies addressing RQ1 and RQ2 were required to report machine learning predictive models relevant to healthcare service quality outcomes, including evidence on model performance, algorithm comparison, accuracy, or validation. In contrast, qualitative studies and studies without predictive model evaluation were eligible only if they contributed evidence relevant to RQ3, such as implementation challenges, clinical integration, or practical implications for decision making. These studies were not included in comparisons of predictive model performance.

The full-text exclusion categories were defined as mutually exclusive and applied hierarchically. Studies were first excluded if they did not address healthcare service quality prediction with machine learning (RQ1) (*n* = 35). Studies remaining after this step were then excluded if they did not report algorithm comparison, performance, accuracy, validation, or other evidence relevant to RQ2 (*n* = 54). Finally, studies were excluded if they did not address practical implications for healthcare application, clinical integration, or decision making (RQ3) (*n* = 44). Each excluded study was assigned to only one category according to the primary reason for exclusion. A final total of 49 studies was included in the synthesis, and all met the predefined inclusion criteria. This structured selection process enhanced transparency and reproducibility.

### 2.3. Data Analysis

As part of the initiatives to assess the applicability of ML applications for the prediction of healthcare quality, a strict data retrieval protocol was utilized with the help of Microsoft Excel (v.2024). For each research study included, the researchers systematically collected and comprehensively indicated the following: author information, publication year, and geographic context as background to this research. The data that were drawn out were first subjected based on three theoretical perspectives of this research that were employed: (a) finding the appropriate healthcare service quality indicators responsible for a machine learning algorithm’s adjustment (e.g., length of stay, readmission rates, and patient experience scores); (b) evaluating the algorithms’ performance in various machine learning configurations (e.g., random forests, support vector machines, and neural networks); and (c) analyzing the practical issues and challenges in understanding implementation, such as model interpretability, data quality issues, and clinical integration barriers. Using this method was beneficial as it enabled the planned simultaneous introduction of the extracted data to the survey’s objectives and the preservation of the methodological process for replicability.

Furthermore, particular attention was paid to the feature selection techniques and validation strategies. The authors reported, in addition to the ones that were specifically suggested for the predictive modeling approaches, the measurement protocols, such as healthcare settings and outcome measurement strategies, among others, that were detailed. Highlighting the significant features of the 49 studies grouped in this systematic review, which is summarized in Table A3 (Appendix A), the overview outlines each study’s characteristics and predictive frameworks, and the key findings of the included studies. For every reported outcome, the model’s target time, prediction horizon, and evaluation time point were extracted, as outcomes bearing the same label are not necessarily comparable without these parameters. This synthesis allowed direct comparison of the methods and the outcomes in the selected literature.

### 2.4. Quality Appraisal and Risk of Bias Assessment

Methodological quality and risk of bias for all included prediction model studies were independently assessed by two reviewers using the PROBAST (Prediction Model Risk of Bias Assessment Tool) framework [27]. Disagreements were resolved through discussion and, where necessary, consultation with a third reviewer. Risk of bias and applicability concerns were evaluated across the four PROBAST domains—Participants, Predictors, Outcome, and Analysis—covering all 20 signaling questions [27]. Particular attention was given to the appropriateness of data sources and study design, predictor handling, potential data leakage during preprocessing, effective sample size, missing-data handling, and internal and external validation procedures. Non-predictive survey and qualitative studies contributing contextual evidence for RQ3 were not appraised using PROBAST because they did not involve prediction model development or validation. The detailed study- and domain-level appraisal results are presented in Table A2 (Appendix A).

## 3. Results

### 3.1. Characteristics of the Included Studies

As evidenced in Table 2, the studies that were included were different in terms of their geographical focus, publication time, methodology, quality metrics, and machine learning (ML) algorithms employed. The majority of studies (55.0%; *n* = 27) were executed in North America, followed by the Asian region (23.0%; *n* = 11) and the European region (18.0%; *n* = 9). Only a small number originated from Oceania and Africa (2.0% each; *n* = 1), which may reflect a possible lack of representation in the literature of low- and middle-income regions. The greatest number of experiments were obtained in 2025 (25.0%; *n* = 12), followed by 2022 and 2020 (16.0% and 17.0%, respectively). A slight decline was seen in 2024 (12.0%, *n* = 6), which could possibly be due to delays in publication or data collection. The upward inclination towards 2025 indicates that the utilization of ML in healthcare quality assessment is more and more popular. The majority of the included studies employed retrospective cohort or observational designs (80.0%; *n* = 39), while cross-sectional, survey-based, and content analysis methodologies accounted for 12.0% (*n* = 6). Only 8.0% (*n* = 4) evaluated models within prospective cohort or quasi-experimental frameworks. This distribution underscores a strong reliance on pre-existing, historical datasets rather than prospective, real-time clinical evaluations. For descriptive purposes, studies were assigned to one mutually exclusive algorithm category at the study level based on the primary model class analyzed in each article. Therefore, the ML algorithm percentages shown in Table 2 reflect study-level classification rather than the total frequency of use of all individual models across the included studies.

The most commonly evaluated metric was the index of the length of stay (LOS) (27.0%; *n* = 13), followed by mortality (23.0%; *n* = 11) and readmission rates (18.0%; *n* = 9). Resource utilization and cost were examined in 16.0% (*n* = 8) of studies, whereas wait times (8.0%; *n* = 4) and patient experience/satisfaction (6.0%; *n* = 3) were less common. Τhis indicates a predominant focus on operational and clinical outcomes over patient-reported measures [28,29,30,31,32]. However, broader survey-based evidence highlights that healthcare stakeholders view machine learning integration as a vital strategic asset for overall service quality and operational resource optimization [30,32].

At the study level, 32 studies (65.0%) were classified as primarily evaluating traditional or ensemble ML methods, whereas 17 studies (35.0%) were classified as primarily evaluating deep learning or neural network approaches. This categorization was used for descriptive summary purposes and should not be interpreted as the total frequency of use of individual algorithm families across all comparative analyses.

### 3.2. Quality Appraisal Findings

Quality appraisal of the 46 prediction model studies using PROBAST [27] indicated that most studies had a low overall risk of bias and low applicability concerns (Table A2). Overall, 33 studies were rated as low risk of bias, 11 as high risk of bias, and two as unclear risk of bias, while applicability concerns were uniformly low across the participant, predictor, and outcome domains. Methodological limitations were concentrated primarily in the analysis domain, where 10 studies were rated as high risk and two as unclear risk, most commonly due to insufficient reporting of validation procedures, limited calibration assessment, and weaknesses in analytic handling during model development and evaluation. Two studies were additionally rated as high risk in the participants domain, which also contributed to their overall high-risk classification. These appraisal findings were taken into account in the narrative synthesis, with greater interpretive weight given to studies judged to be methodologically robust and well validated.

### 3.3. Main Healthcare Service Quality Metrics Targeted by ML Models

The 49 included studies evaluated a diverse range of healthcare service quality metrics across various clinical and operational settings, consistent with the multidimensional framework outlined in the Introduction. Machine learning models were evaluated on their capacity to predict these interdependent dimensions. To align with this framework, the targeted metrics are presented under three separate subheadings: clinical impact, operational impact, and predictive performance.

#### 3.3.1. Clinical Impact

Predicting Mortality: Mortality was a frequently studied metric, serving as the primary focus in 11 studies [33,34,35]. Researchers developed models for high-stakes environments such as intensive care units (ICUs) and severe trauma centers [36,37,38,39]. Furthermore, studies highlighted specialized ML applications for predicting outcomes in COVID-19 and sepsis, where early prediction is critical [40,41,42,43].

Readmission Outlines as Indicators of Hospital Interventions: Thirty-day readmission, defined as patients returning to the hospital within 30 days of discharge due to the same or related medical condition, was studied in multiple studies [44,45,46,47]. Customized ML models were developed specifically for vulnerable populations, such as diabetic or elderly patients, who have a high rate of hospitalization [48,49,50]. These models integrated specific comorbidity data with medical history to arrive at personalized predictions [51,52].

Patient-Centered and Specialized Metrics: Five studies focused on specialized applications, extending beyond traditional clinical markers. ML models were utilized to predict outcomes for traumatic brain injuries, track medication adherence, and evaluate quality of life [53]. Patient experience and satisfaction were the focus of three studies, employing sentiment analysis for care enhancement [54,55,56,57,58]. Applications ranged from ward bed occupancy management to ensuring quality assurance in specific treatments [44,59,60,61]. Real-world implementation of these models is often associated with similar difficulties in all these metrics [18,50,61].

#### 3.3.2. Operational Impact

Length of Stay (LOS) Optimization: Besides mortality, another critical factor is the healthcare system’s ability to run at the highest possible efficiency in order to be able to serve more patients [62,63,64]. LOS was highlighted in 13 studies. LOS optimization addresses both hospital bed capacity and minimizing unnecessary patient exposure to the hospital environment [65,66]. Implementations ranged from forecasting regular ward admissions to specialized areas like child medicine and ICUs, including predicting exact discharge readiness [35,67,68]. Delay in discharge was addressed by predicting the exact time when a patient is ready for discharge and safe to go home [69,70,71,72].

Resource Allocation and Operational Flow: Twelve studies demonstrated ML’s role in predicting healthcare costs and identifying patients requiring intensive care [73,74]. These models often emerged as more effective when incorporating social determinants of health alongside clinical data [75,76]. Artificial Intelligence (AI) was applied in emergency departments to forecast wait times and manage patient flow, and in surgical departments to estimate operation duration [77,78,79].

#### 3.3.3. Predictive Performance

Comparative Effectiveness of Different ML Algorithms: At the study level, 32 studies were classified as primarily evaluating traditional or ensemble ML methods, whereas 17 studies were classified as primarily evaluating deep learning or neural network approaches. This study-level categorization is descriptive and does not represent the total frequency of use of individual algorithms across all included analyses.

The Principle of Ensemble Methods: Among ensemble methods, random forest (RF) was frequently reported across different clinical prediction tasks [35,37,49,53,64,67,70,71,74]. In ICU settings, RF models achieved AUC values ranging from 0.881 to 0.945 for mortality prediction [34,39,44,63,73]. In trauma-related applications using the Synthetic Minority Over-Sampling Technique (SMOTE), the reported mortality prediction performance exceeded an AUC of 0.91 [33,72]. RF was also reported in pediatric length-of-stay prediction, with reported R^2^ values of up to 0.657, and in emergency department triage, with a reported accuracy of 89.1% [36,67]. Gradient boosting approaches were also reported across several use cases [45,53,74]. XGBoost was used in tasks such as delayed discharge prediction [28,46,76], where one study reported a recall of 0.95 and an AUC of 0.97 [72,73,76], and in surgical duration prediction, where reported mean absolute error (MAE) values ranged from 10.87 to 13.61 min. Mortality prediction studies using gradient boosting approaches reported AUROC values between 0.80 and 0.88 [28,35,44,52,56,66,67]. CatBoost was reported for 30-day readmission prediction in high-risk patient groups, with an AUROC of around 0.79 [28,38,66]. Gradient boosting machines (GBMs) were also applied to healthcare cost prediction, with a reported performance of R^2^ = 0.388 and C-statistic = 0.717 [43,48,56,78].

The Contribution of Traditional Models: Traditional models also remained represented in the included studies. Support vector machines (SVMs) were reported for clinical deterioration detection, with a sensitivity of 81.6% and an AUC of 0.85 [46,69,70,74]. Furthermore, logistic regression (LR), which was an evaluation reference in some research studies, remains an effective and interpretable model [39,45,53,58,76]. When used together with new methods, it was able to reach AUROC values above 0.80 when predicting high healthcare utilization [47,58,60,63,70,71,75].

Deep Learning for Time-Series Analysis: Among deep learning approaches, recurrent architectures such as recurrent neural networks (RNNs), gated recurrent units (GRUs), and long short-term memory (LSTM) networks were reported for time-series prediction tasks, particularly in ICU settings [40,63,73,78]. In one study, these models achieved AUC values ranging from 0.635 to 0.870 [45] across mortality, prolonged length of stay, and readmission outcomes. Artificial neural networks (ANNs) were also reported for hospital length-of-stay prediction, with R^2^ values ranging from 0.807 to 0.854 [33,38,55,66,69].

Interpretability vs. Performance: The literature suggests that ensemble methods like RF and XGBoost, as well as deep learning models, often achieve high predictive performance but face implementation limitations [36,74]. Model complexity does not necessarily translate into better clinical utility [28,47,76]. The results indicate that model success depends not only on the algorithm used, but is also strongly influenced by feature selection, pre-processing, and well-balanced data [42,44,65,71,73]. Because the included studies varied widely in population, outcome, sample size, validation design, and performance metric, no pooled comparative estimate was calculated. Metrics such as AUC, AUROC, R^2^, accuracy, recall, and MAE are not directly comparable across different prediction tasks. Accordingly, algorithm performance should be interpreted within the specific clinical and methodological context of each study.

### 3.4. Literature Findings on Practical Implications and Limitations

The included studies identified several recurring challenges in the implementation of ML models in healthcare settings, particularly related to interpretability, data integration, workflow compatibility, fairness, and ongoing validation [15,41,54].

Interpretation Difficulties in Clinical Implementation: Interpretability was frequently reported as a barrier to clinical implementation [34,48,61,80]. More complex models, particularly some deep learning approaches, were described as having limited interpretability, which can reduce clinician confidence in individual predictions [39,55,72,81]. Studies also reported the use of interpretability methods such as SHAP and attention mechanisms to improve transparency in applied settings [20,37,44,53,66,67,82].

Feature Selection and Predictive Model Efficiency: Feature selection and pre-processing were also reported as important determinants of model performance and implementation feasibility [45,51,61,65]. Several studies identified prior healthcare utilization, comorbidity profiles, and demographic variables as strong predictors across quality metrics, and some reports suggested that smaller feature sets could achieve performance similar to more complex approaches in specific contexts [28,40,41,54]. These findings indicate that performance differences may reflect data preparation and feature engineering as much as algorithm class [28,38].

EHR Integration and Cultivation of Clinical Practice: A machine learning (ML) model, which is exceedingly interpretable, is going to be a disaster if it is not consistent with the normal daily running of a hospital [51,65]. Integration into existing hospital systems remains a recurrent practical limitation [80,81]. The attached review reports that ML integration into Electronic Health Record (EHR) systems is still a technical and organizational challenge, particularly in the presence of heterogeneous data sources and variable data quality [45,61,79]. Implementation studies emphasized data standardization and adaptation of clinical workflows to support ML-based decision processes [14,41].

Algorithmic Fairness and Model Validation: Fairness and generalizability were additional concerns reported in the literature [20,55]. This section of studies describes the risk that models trained on historically biased data may reproduce existing disparities, and it notes that performance may not transfer across hospitals, populations, or time without revalidation [39,43,74,80,82]. Ongoing monitoring, fairness assessment, and external validation, therefore, remain necessary conditions for broader clinical adoption [34,64].

The Blueprint for Success and Real-World Impact: Reported patient-outcome effects should be interpreted cautiously [48,54,70]. In specific real-world implementation studies, ML-supported interventions were associated with reported mortality reductions ranging from 1.9% to 39.5%, but these findings came from heterogeneous study designs and clinical contexts and do not establish a general causal effect of ML on mortality [37,40,42,66]. More consistently, the included studies suggest associations with earlier identification of at-risk patients and with changes in triage, resource allocation, and workflow, which should be distinguished from direct evidence of improved patient outcomes [57,74].

## 4. Discussion

This systematic review is a comprehensive synthesis of the results from 49 recent studies that have explored the use of machine learning (ML) algorithms in forecasting healthcare service quality metrics. By thoroughly examining the existing literature, this research not only highlights the potential of ML-based predictive tools, but also identifies persistent clinical, operational, and ethical challenges encountered in their implementation at scale. The main finding of the report is the shift from designing algorithms to the stage of their deployment in real-world clinical settings [12,81]. Qualitative and non-predictive studies contributed only to the synthesis of implementation barriers, ethical considerations, and practical implications and were not included in comparisons of predictive model performance.

The targeted healthcare quality metrics revealed by the researchers show a clear emphasis in our synthesis, which identified the most common acute clinical and operational outcomes, such as mortality, length of stay (LOS), and hospital readmission rates [48,63,71]. This emphasis likely reflects the immediate financial and life-saving pressures of contemporary acute care environments, as these outcomes are closely linked to operational planning and patient safety [58,66,68]. Just like previous systematic reviews outlining clinical outcomes, the main way to predict these high-risk incidents is through administrative claims data and Electronic Health Records (EHRs) [16,83,84,85]. In addition to this, a number of the articles in this review also managed to introduce predictive modeling beyond general hospital measurements into very specialized clinical pathways [49,71,73]. For instance, ML models could track the progress of multidimensional physiological and psychological patients in cardiac rehabilitation programs to predict clinical deterioration and to forecast even multicomponent postoperative pain and health-related quality of life [29,49,86].

Nevertheless, even with the significant trends in the clinical area, this review underlines that under-represented areas, such as the patient experience, communication quality, and overall satisfaction, remain considerably less represented in the predictive modeling [53,87]. Apart from this, CIRMQUAL, which has become the standard, asserts that the quality of healthcare service is not one-dimensional, but a multidimensional combination of relational and managerial factors, as well as clinical safety and infrastructure that together make up service provision quality [2,6,17,21,40]. Just like the systematic reviews by other researchers, this likely reflects that the lack of this research is not due to disinterest on the clinical side, but rather the significant difficulties involved in unstructured data processing [83,86,87,88]. Analyzing patient recommendations, processing emotional analysis, and managing star ratings call for advanced natural language processing (NLP) methods to handle linguistic complexity, sentiment, and context, as opposed to the relatively straightforward task of assessing structured numerical data such as blood pressure or admission times [86,87]. This remains a major challenge for healthcare systems, which want to comment on the overall quality of services, not only in terms of throughput and survival rates [83,88].

Overall, the results uncover a trade-off between pure predictive accuracy and clinical implementation complexity during the evaluation of the comparative effectiveness of various machine learning algorithms [36,64,75]. Based on the acquired literature, deep learning and neural network models (for instance, CNNs, RNNs, and LSTMs) were always among the top performers with the highest metrics, including AUROC values of 0.99 in some particular critical care use cases [63,78]. These models were the only ones that could manage the continuous and complex time-series data like ICU monitoring [34]. However, this finding aligns with several recent methodological reviews confirming that substantial data requirements, infrastructural demands, and high computational costs represent the primary barriers to large-scale implementation [85,89,90,91].

This review aligns with the scientific consensus that tree-based ensemble methods, especially RF and XGBoost, frequently provide a favorable balance between high performance and clinical feasibility [36,44]. These traditional models often matched deep learning in accuracy but had the advantage of being computationally simple and not likely to overfit on a small dataset [71,78]. Furthermore, not only straightforward models such as SVM but also the newest type of penalized logistic regression (LR) remain effective and sensitive diagnostic instruments [81]. The crucial message is that the influence of pre-processing, feature selection, sample size, and validation strategy may be more important than the algorithm family itself [21,92,93]. Reiterating hyper-specialized investigations, simply designed models can get close to the accuracy of very complex digital designs; that is, by using the minimum computation power, they use accurate specific variables, for instance, specific comorbidities or social health determinants [91,94].

Lastly, the examination of the practical implications and limitations reported across the 49 studies shows that translating these predictive algorithms from the research laboratory to the hospital ward is full of difficulties, both methodological and ethical [58,80]. A primary barrier impeding immediate clinical deployment is the lack of interpretability inherent in complex machine learning architectures [37,66]. Medical experts are duty-bound to comprehend the logic behind the choices of code in medical issues; hence, there is no clinical significance in having better accuracy metrics if the doctor is unable to interpret the use of the algorithm to flag a patient for delayed discharge or high mortality risk [34,79]. Consistent with prior methodological reviews and risk-of-bias assessments, our findings highlight a critical need for robust explanatory frameworks [13,71,92,93]. For example, Shapley additive explanations (SHAP) and attention mechanisms can help make models transparent and gain trust clinically [39,55].

Moreover, a concerning trend across the reviewed literature is that the vast majority of predictive models remain confined to the development or internal validation stage [95]. The prospective application of such models in real-world, clinical settings is alarmingly infrequent. The non-availability of external validation is one of the reasons brought to light for the aforementioned crisis of algorithmic fairness and generalizability [69]. The models that are developed using a dominantly urban and highly localized dataset commonly experience performance degradation or complete failure in setups using diverse rural actors or different demographics in the healthcare system [38,56,81]. If historical patient datasets reflect systemic biases, such as unequal delivery of care across specific demographics, machine learning models risk codifying and perpetuating these disparities, ultimately leading to biased clinical decisions that exacerbate health inequities [94,95]. ML-based solutions can improve predictive discrimination and support clinical workflow functions such as triage and resource allocation in real-world settings, but evidence of better patient outcomes remains context-specific, and causal claims about improved care should be limited to the individual implementation studies that directly evaluated such effects [90,91]. Accordingly, predictive performance metrics should be distinguished from workflow effects, and both should be distinguished from outcome improvements demonstrated in implementation studies.

Translating ML models into clinical practice requires more than high predictive performance on historical, inaccessible datasets [38,84,95]. Several key factors are necessary for effective implementation. The first element is the agreement of academic institutions and the medical sector on the adoption of clear validation protocols [83,89]. The second consideration is the straightforward and seamless merging of numerous Electronic Health Records (EHRs) [16,45]. The third factor is a deliberate commitment throughout the algorithm design process to prevent biased outcomes [92]. Finally, medical practitioners need long-term training and adaptive support [55,85]. These conclusions should be interpreted in light of the methodological quality of the included prediction model studies, as greater weight was given to studies with low risk of bias and stronger validation procedures, while findings from studies with higher or unclear risk of bias were interpreted more cautiously. Only through focused, multidisciplinary collaboration can machine learning transition from an experimental computational tool into a trusted partner in healthcare delivery.

## 5. Limitations and Strengths

This systematic review has several critical strengths. Methodologically, it closely followed PRISMA guidelines and covered a very current timeframe (2020–2025), accordingly reflecting the newest machine learning applications in healthcare. The dominant edge of the literature synthesized lies in its solid assessment of critical outcomes, like readmission, length of stay, and mortality, by means of large-scale datasets sourced predominantly from intensive care units (ICUs) and emergency departments. Furthermore, by systematically contrasting algorithmic performance with real-world implementation challenges, this review provides a comprehensive, actionable perspective that extends beyond a purely technical evaluation of predictive models.

Nevertheless, some limitations should be accepted, both in the literature reviewed and in the review itself. To start with the studies synthesized, a serious gap is the lack of external validation of predictive models among various populations and healthcare systems, thus undermining both geographic and demographic generalizability. Moreover, there is a definite typification of outpatient settings and an alarming absence of rigorous algorithmic equity and bias analyses, particularly regarding metrics that are subjective, such as wait times and patient experience.

With regard to the review itself, a notable limitation was the high non-retrieval rate at the full-text screening stage. Of the 404 records sought for retrieval, 222 could not be accessed despite the use of multiple access strategies, which may have introduced availability bias and reduced the comprehensiveness of the final evidence base. This limitation should be considered when interpreting the completeness and generalizability of the review findings. In addition, the literature search was confined to articles published in English, which may have introduced language bias. Lastly, publication bias remains a possible concern, as studies reporting stronger predictive performance are more likely to be published than studies with poor or null results.

## 6. Conclusions

This close examination of 49 studies proves that ML algorithms, especially ensemble methods and deep learning architectures, have the ability to predict multidimensional healthcare service quality metrics very well. By predicting critical outcomes such as mortality, length of stay, and readmission rates with high accuracy, these tools have become unprecedented methods for risk stratification, operational efficiency and targeted resource allocation.

However, the transition from theoretical aspects of algorithm development to actual practical application in the medical field is still opposed by many essential methodological, technical and ethical challenges. The persistent opacity of these complex models, combined with a lack of prospective external validation, severely undermines clinical trust and risks amplifying existing healthcare disparities through biased algorithmic outputs.

Moreover, the existing bibliography’s inconsistent focus on acute clinical metrics indicates the urgent necessity to expand the area of ML application to patient-centered domains, like patient experience and satisfaction. The later stage of machine learning in healthcare quality assessment is not the insistent pursuit of a minor increase in the correctness of prediction on separate, retrospective datasets. The most realistic way to make a huge difference in this world is to move toward algorithmic transparency. Explainable AI (XAI) is the knowledge that allows humans to interpret and build their trust in the output results delivered by machine learning algorithms.

Even so, the achievement of clinical adoption also requires standardized external validation frameworks, easy EHR integration, and the collaboration of different disciplines. Only when these primary obstacles are addressed can machine learning transition from a speculative computational experiment into a reliable, equitable, and integral partner in healthcare delivery.

## Figures and Tables

**Table 1:** Conceptual search framework.

Concept Block	Representative Terms
Machine learning	Machine learning; deep learning; artificial intelligence; random forest
Healthcare service quality	Healthcare quality; quality of care; service quality; length of stay; readmission; patient satisfaction; mortality; waiting time
Prediction	Prediction; predictive models; forecasting; prognostic models

**Table 2:** Summary of included studies’ characteristics.

Characteristics	Studies *n* (%)
Region of studies	
Europe	9 (18.0)
Asia	11 (23.0)
North America	27 (55.0)
Oceania	1 (2.0)
Africa	1 (2.0)
Year of publication	
2020	8 (17.0)
2021	7 (14.0)
2022	8 (16.0)
2023	8 (16.0)
2024	6 (12.0)
2025	12 (25.0)
Type of studies	
Retrospective cohort/observational	39 (80.0)
Cross-sectional/survey-based	6 (12.0)
Prospective cohort/quasi-experimental	4 (8.0)
Common quality metrics	
Readmission rates	9 (18.0)
Length of stay (LOS)	13 (27.0)
Mortality	11 (23.0)
Wait times	4 (8.0)
Patient experience and satisfaction	3 (6.0)
Resource utilization and cost	8 (16.0)
Other	1 (2.0)
ML algorithms *	
Deep learning/neural network approaches (CNN/LSTM, etc.)	17 (35.0)
Traditional ML methods (Tree-based, linear, SVM, random forest, gradient boosting, logistic regression, etc.)	32 (65.0)

* Note: The ML algorithm category was coded at the study level according to the primary model class analyzed in each article. Percentages do not represent the total frequency of use of all individual models across this review.

## Data Availability

No new data were created or analyzed in this study. Data sharing is not applicable to this article.

## Supplementary Materials

The following supporting information can be downloaded at https://www.mdpi.com/article/10.3390/healthcare14182887/s1. Table S1: PRISMA 2020 Main Checklist; Table S2: PRISMA 2020 for Abstracts Checklist. Reference [96] is cited in the supplementary materials.

## Abbreviations

The following abbreviations are used in this manuscript:

AI	Artificial Intelligence
ANN	Artificial Neural Network
AUC	Area Under the Curve
AUROC	Area Under the ROC Curve
CNN	Convolutional Neural Network
DL	Deep Learning
DT	Decision Tree
ED	Emergency Department
GBM	Gradient Boosting Machine
GRU	Gated Recurrent Unit
ICU	Intensive Care Unit
KNN	K-Nearest Neighbors
ML	Machine Learning
LOS	Length of Stay
LR	Logistic Regression
LSTM	Long Short-Term Memory
MAE	Mean Absolute Error
NB	Naïve Bayes
NLP	Natural Language Processing
RF	Random Forest
RNN	Recurrent Neural Network
ROC	Receiver Operating Characteristic
SHAP	Shapley Additive Explanations
SMOTE	Synthetic Minority Over-Sampling Technique
SVM	Support Vector Machine
XAI	Explainable AI
XGBoost	Extreme Gradient Boosting

## Appendix A

**Table A1:** Full database-specific search strings, syntax, field restrictions, and execution dates.

Database	Date of Search	Search Strings—Syntax	Filters/Limits	Records
PubMed/MEDLINE	15 July 2025	(“machine learning”[MeSH Terms] OR “machine learning”[Title/Abstract] OR “deep learning”[Title/Abstract] OR “artificial intelligence”[MeSH Terms] OR “artificial intelligence”[Title/Abstract] OR “random forest”[Title/Abstract] OR “gradient boosting”[Title/Abstract] OR “neural network*”[Title/Abstract] OR “support vector machine*”[Title/Abstract]) AND (“healthcare quality”[Title/Abstract] OR “quality of healthcare”[MeSH Terms] OR “service quality”[Title/Abstract] OR “quality metrics”[Title/Abstract] OR “length of stay”[MeSH Terms] OR “length of stay”[Title/Abstract] OR “patient readmission”[MeSH Terms] OR “hospital readmission”[Title/Abstract] OR “patient satisfaction”[MeSH Terms] OR “patient satisfaction”[Title/Abstract] OR “hospital mortality”[MeSH Terms] OR “mortality”[Title/Abstract] OR “waiting time*”[Title/Abstract]) AND (“prediction”[Title/Abstract] OR “predictive model*”[Title/Abstract] OR “forecasting”[MeSH Terms] OR “forecasting”[Title/Abstract] OR “prognos*”[Title/Abstract])	Years: 1 January 2020–30 July 2025; language: English; species: humans; article type: original journal articles	534
Scopus (Elsevier)	20 July 2025	TITLE-ABS-KEY ((“machine learning” OR “deep learning” OR “artificial intelligence” OR “random forest” OR “gradient boosting” OR “neural network*” OR “support vector machine*”) AND (“healthcare quality” OR “service quality” OR “quality of care” OR “quality metric*” OR “length of stay” OR “readmission” OR “patient satisfaction” OR “mortality” OR “wait* time*”) AND (“predict*” OR “forecasting” OR “prediction model*” OR “prognostic”))	PUBYEAR > 2019 and PUBYEAR < 2026; language: English; DOCTYPE: “ar” (article); SRCTYPE: “j” (journal)	204
CINAHL (EBSCOhost)	25 July 2025	((MH “Machine Learning+”) OR TI (“machine learning” OR “deep learning” OR “artificial intelligence” OR “neural network*” OR “random forest”) OR AB (“machine learning” OR “deep learning” OR “artificial intelligence” OR “neural network*” OR “random forest”)) AND ((MH “HealthCare Quality+”) OR TI (“service quality” OR “quality metric*” OR “length of stay” OR “readmission” OR “patient satisfaction” OR “mortality” OR “wait* time*”) OR AB (“service quality” OR “quality metric*” OR “length of stay” OR “readmission” OR “patient satisfaction” OR “mortality” OR “wait* time*”)) AND ((MH “Predictive Analytics”) OR (MH “Predictive Modeling”) OR TI (predict* OR forecast* OR “risk model*”) OR AB (predict* OR forecast* OR “risk model*”))	Published date: 1 January 2020–30 July 2025; peer-reviewed; language: English; exclude MEDLINE records to avoid duplicate overlap	187
ScienceDirect (Elsevier)	30 July 2025	Title, abstract, keywords: (“machine learning” OR “deep learning” OR “artificial intelligence”) AND (“healthcare service quality” OR “length of stay” OR “hospital readmission” OR “patient satisfaction”) AND (“prediction” OR “forecasting”)	Years: 2020–2025; article type: research articles; subject areas: medicine and dentistry, computer science, and health professions	125

‘*’ denotes the truncation wildcard used in database search queries to capture word variants (e.g., singular and plural forms).

**Table A2:** PROBAST (Prediction Model Risk of Bias Assessment Tool)/risk-of-bias and applicability assessment.

A/A	Study (Author, Year)	Risk of Bias				Applicability			Overall	
		Participants	Predictors	Outcome	Analysis	Participants	Predictors	Outcome	Risk of Bias	Applicability
1	Abakasanga et al., 2025 [71]	Low	Low	Low	Low	Low	Low	Low	Low	Low
2	Ameur et al., 2022 [35]	Low	Low	Low	Unclear	Low	Low	Low	Unclear	Low
3	Avinash et al., 2025 [62]	Low	Low	Low	Low	Low	Low	Low	Low	Low
4	Bari et al., 2020 [53]	Low	Low	Low	Low	Low	Low	Low	Low	Low
5	Boussina et al., 2024 [40]	Low	Low	Low	Low	Low	Low	Low	Low	Low
6	Boutry et al., 2024 [73]	Low	Low	Low	High	Low	Low	Low	High	Low
7	Burdick et al., 2020 [41]	Low	Low	Low	Low	Low	Low	Low	Low	Low
8	Davis et al., 2022 [44]	Low	Low	Low	Low	Low	Low	Low	Low	Low
9	Deng et al., 2022 [63]	Low	Low	Low	Low	Low	Low	Low	Low	Low
10	Earnest et al., 2023 [64]	Low	Low	Low	Low	Low	Low	Low	Low	Low
11	Elhaj et al., 2023 [36]	Low	Low	Low	Unclear	Low	Low	Low	Unclear	Low
12	Emi-Johnson and Nkrumah, 2025 [45]	Low	Low	Low	High	Low	Low	Low	High	Low
13	Fan et al., 2025 [60]	Low	Low	Low	Low	Low	Low	Low	Low	Low
14	Farhat et al., 2024 [74]	Low	Low	Low	Low	Low	Low	Low	Low	Low
15	Fu et al., 2025 [39]	Low	Low	Low	Low	Low	Low	Low	Low	Low
16	Grim et al., 2024 [59]	High	Low	Low	High	Low	Low	Low	High	Low
17	Hall et al., 2023 [75]	Low	Low	Low	Low	Low	Low	Low	Low	Low
18	Hassanzadeh et al., 2023 [33]	High	Low	Low	High	Low	Low	Low	High	Low
19	Hernández-Arango et al., 2025 [76]	Low	Low	Low	Low	Low	Low	Low	Low	Low
20	Hilton et al., 2020 [48]	Low	Low	Low	Low	Low	Low	Low	Low	Low
21	Irvin et al., 2020 [56]	Low	Low	Low	Low	Low	Low	Low	Low	Low
22	Iwase et al., 2022 [34]	Low	Low	Low	Low	Low	Low	Low	Low	Low
23	Jaotombo et al., 2023 [65]	Low	Low	Low	Low	Low	Low	Low	Low	Low
24	Kia et al., 2020 [49]	Low	Low	Low	Low	Low	Low	Low	Low	Low
25	Kiremit and Yardan, 2025 [66]	Low	Low	Low	Low	Low	Low	Low	Low	Low
26	Koesmahargyo et al., 2020 [54]	Low	Low	Low	High	Low	Low	Low	High	Low
27	Langenberger et al., 2025 [77]	Low	Low	Low	Low	Low	Low	Low	Low	Low
28	Lewis et al., 2021 [78]	Low	Low	Low	Low	Low	Low	Low	Low	Low
29	Li et al., 2020 [51]	Low	Low	Low	Low	Low	Low	Low	Low	Low
30	MacKay et al., 2021 [79]	Low	Low	Low	Low	Low	Low	Low	Low	Low
31	Matsuo et al., 2020 [37]	Low	Low	Low	High	Low	Low	Low	High	Low
32	Medeiros et al., 2025 [67]	Low	Low	Low	Low	Low	Low	Low	Low	Low
33	Michailidis et al., 2022 [46]	Low	Low	Low	Low	Low	Low	Low	Low	Low
34	Mohanty et al., 2022 [47]	Low	Low	Low	Low	Low	Low	Low	Low	Low
35	Moulaei et al., 2022 [42]	Low	Low	Low	High	Low	Low	Low	High	Low
36	Pahlevani et al., 2025 [72]	Low	Low	Low	Low	Low	Low	Low	Low	Low
37	Pfohl et al., 2021 [58]	Low	Low	Low	Low	Low	Low	Low	Low	Low
38	Ravaut et al., 2021 [50]	Low	Low	Low	Low	Low	Low	Low	Low	Low
39	Ricciardi et al., 2024 [68]	Low	Low	Low	High	Low	Low	Low	High	Low
40	Ricket et al., 2022 [57]	Low	Low	Low	Low	Low	Low	Low	Low	Low
41	Tan et al., 2024 [55]	Low	Low	Low	Low	Low	Low	Low	Low	Low
42	Wang et al., 2025 [69]	Low	Low	Low	Low	Low	Low	Low	Low	Low
43	Wichmann et al., 2023 [38]	Low	Low	Low	Low	Low	Low	Low	Low	Low
44	Xiaoqing et al., 2023 [52]	Low	Low	Low	High	Low	Low	Low	High	Low
45	Yang et al., 2025 [43]	Low	Low	Low	High	Low	Low	Low	High	Low
46	Zalikha et al., 2023 [70]	Low	Low	Low	High	Low	Low	Low	High	Low

Low = low risk of bias/low concern of applicability, High = high risk of bias/high concern of applicability, and Unclear = unclear risk of bias/unclear concern of applicability.

**Table A3:** Research studies by author(s), country, aim, study design, sample size, main statistical metric(s), model’s target time, prediction horizon, and evaluation time point, algorithms(s) compared and key result(s).

A/A	Author(s), Year	Country	Aim	Study Design	Sample Size	Main Statistical Metric(s)	Target Τime	Prediction Horizon	Evaluation Time Point	Algorithm(s) Compared	Key Result(s)
1	Abakasanga et al., 2025 [71]	UΚ	Predict LOS for patients with LD, MLTCs, and addressing ethnic fairness	Retrospective cohort study	9618 patients (62,243 admissions)	AUC-ROC, balanced accuracy, FNR, and FPR	Hospital admission	>7–14-day stay	Hospital discharge	LR, SVM, RF, XGBoost, HistGradientBoosting, KNN, and ANN	RF achieved the best performance with AUC-ROC values of 0.759 (males) and 0.756 (females) and balanced accuracies of 0.690 (males) and 0.689 (females).
2	Ameur et al., 2022 [35]	France	Predict patient waiting times in emergency departments	Retrospective cohort study	88,166 patients	MAE and RMSE	ED triage arrival	Continuous wait time (minutes)	Patient departure/bed placement	RF, LASSO, Huber Regressor, SVR, and DNN	DNN demonstrated the best predictive capability among the evaluated models.
3	Avinash et al., 2025 [62]	India	Forecast weekly bed occupancy in a psychiatric tertiary care hospital	Retrospective time-series study	16-year weekly bed occupancy records	RMSE, MAE, MAPE, and Diebold–Mariano test	Start of forecast week	1 to 4 weeks ahead	End of each targeted week	SVR, XGBoost, RF, KNN, GBDT, and decision tree (DT)	XGBoost significantly outperformed other models, achieving the lowest forecasting errors.
4	Bari et al., 2020 [53]	United States	Predict patient responses to HCAHPS survey “Doctor Communications” questions	Retrospective observational study	216,737 discharges (31,300 survey responses)	AUC-ROC, sensitivity, specificity, PPV, and NPV	Inpatient stay/discharge	Retrospective encounter rating	HCAHPS survey return (30–42 days post-discharge)	RF, LR, and DT	RF outperformed alternative models across all survey domains (AUC-ROCs: 0.819–0.876).
5	Boussina et al., 2024 [40]	United States	Evaluate the clinical impact of a deep learning sepsis prediction model (COMPOSER) on patient outcomes	Quasi-experimental before-and-after study	6217 patient encounters	In-hospital mortality, sepsis bundle compliance, and 72 h SOFA score change	Continuous real-time ICU monitoring	4–6 h prior to sepsis onset	Sepsis onset/in-hospital mortality	Deep learning (COMPOSER)	Implementation resulted in a 1.9% absolute reduction in sepsis mortality and a 5.0% increase in bundle compliance.
6	Boutry et al., 2024 [73]	France	Predict patient-specific quality assurance in breast IMRT radiotherapy plans using complexity metrics	Retrospective analysis	318 beams (56 patients)	AUC-ROC, sensitivity, and specificity	Pre-treatment planning phase	Immediate plan verification failure	Delivery verification completion	ML and DL classifiers	Optimized DL model showed superior performance with an AUC-ROC of 0.95, a sensitivity of 0.97, and a specificity of 0.98.
7	Burdick et al., 2020 [41]	United States	Assess the impact of an ML algorithm for severe sepsis prediction on clinical outcomes	Prospective cohort study	75,147 patient encounters (17,758 sepsis-related)	In-hospital mortality, length of stay (LOS), and 30-day readmission rate	Continuous inpatient monitoring	Prior to clinical decompensation	In-hospital mortality, LOS, and 30-day readmission	Proprietary ML algorithm	Led to a 39.5% reduction in in-hospital mortality, 32.3% reduction in LOS, and 22.7% reduction in 30-day readmission.
8	Davis et al., 2022 [44]	Canada	Develop and evaluate predictive models for hospital readmission using longitudinal automated features	Retrospective cohort study	428,669 patients	AUC-ROC	Hospital discharge	30 days post-discharge	30 days post-discharge	LACE index and gradient boosting machine (GBM)	GBM with learned features achieved an AUC-ROC of 0.83, significantly outperforming the LACE index (AUC-ROC: 0.66).
9	Deng et al., 2022 [63]	United States	Predict in-hospital mortality, prolonged length of stay, and 30-day readmission in intensive care units	Retrospective cohort study	40,083 ICU patients	AUC-ROC, sensitivity, specificity, and Brier score	First 24 h of ICU stay	In-hospital/ICU stay/30 days	ICU/hospital discharge and 30 days post-discharge	RNN, GRU, and LSTM with attention	LSTM with attention achieved an AUC-ROC of 0.870 for mortality, 0.765 for prolonged LOS, and 0.635 for readmission.
10	Earnest et al., 2023 [64]	Australia	Predict the quality and timeliness of multidisciplinary care delivery in lung cancer patients	Retrospective cohort study	11,602 patients	AUC-ROC	Lung cancer diagnosis/MDT referral	≤28–42 days to treatment	Treatment initiation date	RF, KNN, ANN, and SVM	SVM yielded the highest discrimination with an out-of-sample AUC-ROC of 0.89.
11	Elhaj et al., 2023 [36]	United Kingdom	Build an automated triage classification model for predicting emergency department patient disposition	Retrospective comparative study	2688 patients	Micro-accuracy, precision, recall, and F1-score	ED presentation/initial triage	Current ED encounter duration	Inpatient admission vs. discharge	KNN, GBDT, XGBoost, and RF	RF proved optimal, achieving 89.1% micro-accuracy, 89.0% precision, 89.1% recall, and an 89.0% F1-score.
12	Emi-Johnson and Nkrumah, 2025 [45]	United States	Compare four ML models in predicting 30-day hospital readmissions and determine primary risk factors	Retrospective cohort study	101,766 inpatient encounters	Accuracy, precision, recall, F1-score, and AUC-ROC	Hospital discharge	30 days post-discharge	30 days post-discharge	LR, RF, XGBoost, and DNN	XGBoost achieved the best discriminative capacity (AUC-ROC: 0.667); prior admissions and comorbidities were top predictors.
13	Fan et al., 2025 [60]	China	Predict treatment preferences and referral patterns among rehabilitation outpatients	Multicenter cross-sectional study	4244 patients (38 institutions)	Accuracy, precision, recall, F1-score, and AUC-ROC	Initial outpatient evaluation	Rehabilitation episode	Final referral confirmation/discharge	XGBoost, RF, and LR	XGBoost demonstrated the highest overall accuracy (80.21%), closely followed by RF.
14	Farhat et al., 2024 [74]	Tunisia	Predict ambulance transportation requirements in pre-hospital emergency medical services	Retrospective cross-sectional study	93,712 emergency calls	Accuracy, specificity, and sensitivity	Initial emergency dispatch call	Dispatch and pre-hospital transit	Patient transfer to hospital ED	RF, SVM, XGBoost, and AdaBoost	XGBoost yielded the highest accuracy (83.1%) and high specificity (95.39%).
15	Fu et al., 2025 [39]	United States	Predict 30-day mortality and readmission following definitive surgical resection of head and neck cancer	Retrospective cohort study	103,891 patients	AUC-ROC, sensitivity, and specificity	Surgical discharge	30 days post-resection	30 days post-resection	LR, SGD-LR, CatBoost, XGBoost, and stacking classifier	Stacked ensemble achieved AUC-ROC values of 0.80 for 30-day mortality and 0.67 for 30-day readmission.
16	Grim et al., 2024 [59]	United States	Predict health-related quality of life (HRQoL) risk among primary care patients using electronic health records	Prospective cohort study	375 patients	Accuracy, sensitivity, specificity, and balanced accuracy	Primary care visit	Next scheduled clinical check/6–12 months	HRQoL PROMs follow-up evaluation	DT, SVM, RF, ANN, and boosting algorithms	RF and boosting achieved balanced accuracies of 98% for mental HRQoL and 94% for physical HRQoL.
17	Hall et al., 2023 [75]	Canada	Develop an automated machine learning triage acuity prediction model for virtual urgent care	Retrospective cohort study	2,460,109 encounters	Precision, recall, F1-score, and accuracy	Virtual encounter intake	Current virtual consultation	Clinical disposition/in-person referral	DT, KNN, RF, gradient boosting regressor (GBR), and ANN	GBR achieved 47.4% exact acuity agreement and 95.0% safe/up-triaged agreement.
18	Hassanzadeh et al., 2023 [33]	Iran	Compare SMOTE-based ML algorithms for hospital mortality prediction in trauma patients with imbalanced data	Retrospective cohort study	126 ICU trauma patients	Sensitivity, specificity, PPV, NPV, accuracy, AUC-ROC, G-mean, and F1-score	ICU admission (within 24 h)	Entire ICU admission duration	ICU discharge or death	DT, RF, Naïve Bayes (NB), ANN, SVM, and XGBoost	Resampling significantly boosted performance; RF and ANN with SMOTE achieved AUC-ROC > 0.98.
19	Hernández-Arango et al., 2025 [76]	Colombia	Predict adverse outcomes (mortality, hospitalization, and ED visits) in chronic noncommunicable diseases	Retrospective cohort study	4845 patients	AUC-ROC, sensitivity, specificity, PPV, NPV, and calibration	Baseline clinical assessment	12 months follow-up	12 months post-baseline	Elastic Net LR, XGBoost, and ANN	XGBoost achieved superior discrimination for hospitalization (AUC-ROC: 0.963) and mortality (AUC-ROC: 0.896).
20	Hilton et al., 2020 [48]	United States	Develop interpretable ML models for personalized prediction of 30-day readmission, prolonged LOS, and mortality	Retrospective cohort study	1,485,880 hospitalizations (708,089 patients)	AUC-ROC, Brier score, precision, recall, F1-score, and RMSE	Hospital admission and discharge	LOS > 5 days/30-day post-discharge	Hospital discharge and 30 days post-discharge	GBM, DNN, and LR	GBM achieved AUC-ROC values of 0.76 for 30-day readmission and 0.84 for LOS > 5 days with SHAP explainability.
21	Irvin et al., 2020 [56]	United States	Assess ML and area-level social determinants of health (SDH) in prospective risk adjustment for health spending	Retrospective cohort study	1,176,095 adult enrollees	R^2^, mean absolute error (MAE), and C-statistic	Prior annual insurance enrollment	Next calendar year (12 months)	End of subsequent financial year	Ordinary least squares (OLS), LASSO, and LightGBM	LightGBM improved the R^2^ to 0.388 and the C-statistic to 0.717; SDH indicators reduced underpayment in vulnerable groups.
22	Iwase et al., 2022 [34]	Japan	Predict ICU mortality and length of stay categories using routine admission laboratory data	Retrospective cohort study	12,747 ICU patients	AUC-ROC, sensitivity, specificity, and Brier score	ICU admission (routine labs)	ICU stay/≤2 days vs. ≥8 days	ICU discharge or death	RF, XGBoost, ANN, and ordinal forest	RF achieved AUC-ROC values of 0.945 for ICU mortality and 0.881/0.889 for short/long ICU stays; LDH was a key predictor.
23	Jaotombo et al., 2023 [65]	France	Predict prolonged hospital length of stay (>14 days) using administrative medico-administrative data	Retrospective cohort study	73,182 hospitalizations	AUC-ROC and permutation feature importance	Inpatient admission	>14 days stay	Hospital discharge	LR, CART, RF, gradient boosting (GB), and ANN	GB achieved the highest discrimination (AUC-ROC: 0.810), significantly outperforming LR (AUC-ROC: 0.795).
24	Kia et al., 2020 [49]	United States	Develop MEWS++ ML model for early prediction of clinical deterioration or inpatient mortality within 6 h	Retrospective cohort study	96,645 patients (157,984 encounters)	Sensitivity, specificity, accuracy, PPV, F1-score, AUC-ROC, and AUC-PR	Inpatient monitoring	Next 6 to 12 h	6–12 h post-prediction trigger	RF (MEWS++), linear SVM, and LR	RF achieved an AUC-ROC of 0.85 and an AUC-PR of 0.37, significantly outperforming traditional MEWS.
25	Kiremit and Yardan, 2025 [66]	Turkey	Predict hospital length of stay (LOS) across diverse surgical and medical wards using routine service data	Retrospective cohort study	162,140 patients	R^2^, RMSE, and MAE	Inpatient admission	Full inpatient stay (days)	Hospital discharge	ANN (LM, BR, and SCG algorithms), ANFIS-GP, and MLR	Double-hidden-layer ANN-LM achieved the best performance (test R^2^ = 0.807, RMSE = 2.77 days, and MAE = 1.99 days).
26	Koesmahargyo et al., 2020 [54]	United States	Predict dynamic medication non-adherence using smartphone-based computer vision monitoring	Prospective cohort study	4182 clinical trial participants	Accuracy, precision, recall, and AUC-ROC	Daily smartphone intake check	Subsequent 7 days/trial duration	Weekly and final adherence audit	XGBoost	XGBoost achieved AUC-ROC values of 0.83 for study-long adherence and 0.87 for subsequent-week adherence.
27	Kumar et al., 2021 [82]	India	Explore responsible AI constituents in healthcare and analyze impacts on cognitive engagement and market value	Cross-sectional study	12 qualitative interviews, 290 provider surveys, and 174 patient dyads	PLS-SEM path coefficients, R^2^, and model fit indices	Cross-sectional survey	Cross-sectional assessment	Survey completion	Structural equation modeling (PLS-SEM)	Established responsible AI as a third-order factor, validating cognitive engagement as a mediator of perceived value and market performance.
28	Langenberger et al., 2025 [77]	Germany	Predict duration of surgery (DOS) in total hip and knee arthroplasty using preoperative data and PROMs	Retrospective cohort study	3704 patients (multi-hospital); 1815 patients (single-hospital)	MAE, RMSE, and MAPE	Preoperative planning/admission	Intraoperative operating time	Surgical closure/OR exit	XGBoost and multivariable linear regression	XGBoost achieved MAEs of 10.87 min (hip) and 12.53 min (knee) in the single-hospital setting.
29	Lewis et al., 2021 [78]	United States	Compare deep learning and traditional models for predicting preventable acute care utilization and spending in heart failure	Retrospective cohort study	93,260 heart failure patients	Precision at k, cost capture at k, and AUC-ROC	Annual baseline period	Next 12 months	End of follow-up year	LR, enhanced LR, GBM, FNN, CNN, and LSTM with attention	Sequential deep learning (LSTM/CNN) achieved highest AUC-ROCs (0.681–0.778) and captured 30.1% of preventable costs at the top 1%.
30	Li et al., 2020 [51]	Canada	Evaluate ML algorithms for predicting all-cause 30-day hospital readmission at admission and discharge	Retrospective cohort study	1,631,611 hospital stays	AUC-ROC and calibration curves	Inpatient admission and discharge	30 days post-discharge	30 days post-discharge	Penalized LR, Naïve Bayes, RF, deep learning, and XGBoost	XGBoost achieved AUC-ROC > 0.88 at discharge; diagnostic and procedure codes were the strongest predictors.
31	MacKay et al., 2021 [79]	United States	Predict 30-day mortality, rehospitalization, and 23 adverse clinical events from Medicare claims data	Retrospective cohort study	770,777 Medicare beneficiaries	AUC-ROC, Brier score, and sensitivity at alert rate	Hospital discharge/claims date	30 days post-discharge	30 days post-index date	SVM, RF, MLP, XGBoost, and LR	XGBoost achieved AUC-ROC values of 0.88 for mortality and 0.80–0.87 for 17 of 23 adverse events.
32	Matsuo et al., 2020 [37]	Japan	Develop ML models to predict in-hospital morbidity and mortality following non-penetrating traumatic brain injury	Retrospective cohort study	232 TBI patients	Sensitivity, specificity, accuracy, and AUC-ROC	ED/hospital admission	In-hospital stay duration	Hospital discharge or death	Ridge, LASSO, RF, GBDT, extra trees, DT, Gaussian NB, and SVM	RF achieved the best performance for morbidity (AUC-ROC: 0.895) and Ridge regression for mortality (AUC-ROC: 0.875).
33	Medeiros et al., 2025 [67]	Brazil	Predict continuous length of stay in general pediatric inpatient wards using machine learning	Retrospective cohort study	3461 pediatric patients	R^2^, MAE, and RMSE	Pediatric admission	Full admission duration (days)	Hospital discharge	Multiple linear regression, RF, SVR, Ridge, and PLS	RF achieved the best performance, with an R^2^ of 0.657 and an MAE of 3.51 days.
34	Michailidis et al., 2022 [46]	Greece	Forecast 30-day all-cause hospital readmissions integrating clinical, administrative, and operational capacity data	Retrospective cohort study	11,172 hospitalizations	Sensitivity (recall), specificity, precision, accuracy, F1-score, and AUC-ROC	Hospital discharge	30 days post-discharge	30 days post-discharge	Linear SVM, RBF SVM, weighted RF, and balanced RF	Balanced random forest achieved the highest sensitivity (0.70) and an AUC-ROC of 0.78; clinic occupancy was a major predictor.
35	Mohanty et al., 2022 [47]	United States	Predict 30-day readmission risk among older frail adults using multidimensional electronic health records	Retrospective cohort study	128,518 observations (68,152 patients)	Precision, recall, F1-score, and AUC-ROC	Hospital discharge	30 days post-discharge	30 days post-discharge	LR, RF, XGBoost, CatBoost, and stacking classifier	CatBoost achieved an AUC-ROC of 0.79; SHAP analysis highlighted discharge destination, comorbidity index, and frailty flags.
36	Moulaei et al., 2022 [42]	Iran	Compare supervised ML classifiers for predicting in-hospital mortality among confirmed COVID-19 inpatients	Retrospective cohort study	1500 patients	Accuracy, sensitivity, specificity, precision, and AUC-ROC	Hospital admission	In-hospital stay duration	In-hospital death or discharge	J48 DT, MLP, XGBoost, LR, KNN, RF, and Naïve Bayes	RF outperformed other models with 95.03% accuracy, 90.70% sensitivity, 95.10% specificity, and an AUC-ROC of 0.990.
37	Nichol et al., 2021 [80]	United States	Characterize the landscape, target predictions, and organizational traits of commercial predictive analytics in healthcare	Content analysis	106 MLPA products (96 companies)	Descriptive frequency counts; percentage distribution	Product content review	Landscape evaluation	Documented commercial deployment	Categorization of ML algorithms	Identified 5 core prediction categories: disease onset/progression, treatment, cost/utilization, readmissions, and decompensation.
38	Pahlevani et al., 2025 [72]	Canada	Predict alternative level of care (ALC) delayed discharge status at the time of hospital admission	Retrospective cohort study	362,497 hospital stays	Precision, recall, F1-score, specificity, and AUC-ROC	Hospital admission	Inpatient stay until ALC designation	Hospital discharge/ALC transfer	RF, ANN, and XGBoost (with SMOTE, ROS, and class weights)	XGBoost with SMOTE achieved a recall of 0.91 and an AUC-ROC of 0.94 using admission-only features.
39	Pfohl et al., 2021 [58]	United States	Empirically evaluate algorithmic fairness regularization on clinical risk prediction models across large databases	Retrospective cohort study	8,299,385 patients across 3 cohorts (STARR, Optum, and MIMIC-III)	AUC-ROC, average precision, cross-entropy loss, ACE, RCE, and xAUC	Inpatient admission/EHR baseline	30-day/1-year clinical endpoints	End of designated follow-up window	Regularized deep feedforward neural networks	Enforcing group fairness penalties led to significant trade-offs with within-group accuracy and relative calibration.
40	Rahim et al., 2021 [81]	Malaysia	Assess patient-perceived hospital service quality from Facebook reviews	Cross-sectional text-mining study	1825 patient reviews (48 hospitals)	Accuracy, precision, recall, F1-score, and logistic regression OR	Post-visit social review posting	Retrospective service evaluation	Natural language review text analysis	Naïve Bayes, support vector machine, and logistic regression	SVM achieved F1-scores of 0.757 for SERVQUAL dimensions and 0.919 for sentiment classification.
41	Ravaut et al., 2021 [50]	Canada	Predict 3-year risk of adverse outcomes from diabetes complications using population-level administrative data	Retrospective cohort study	1,567,636 diabetes patients	AUC-ROC, calibration curves, and cost capture	Diabetes baseline cohort entry	3 years (36 months)	End of 3-year follow-up period	Gradient boosting decision trees (XGBoost)	XGBoost achieved an average test AUC-ROC of 0.777 across five complication categories and captured 28.1% of costs in the top 5%.
42	Ricciardi et al., 2024 [68]	Italy	Predict prolonged emergency department length of stay (>3 h) using admission workflow characteristics	Retrospective cohort study	496,172 ED visits	Accuracy, precision, recall, and F1-score	ED triage and registration	>3 h in ED	ED discharge or transfer	RF, MLP, Naïve Bayes, LR, and voting ensemble	RF achieved the highest performance with 74.88% accuracy, 72.85% precision, and 74.88% recall.
43	Ricket et al., 2022 [57]	United States	Predict population-level resource-intensive healthcare utilization across hospital service areas (HSAs)	Cross-sectional ecological study	3174 hospital service areas	R^2^, MSE, MAPE, and multiple linear regression	Annual HSA reporting	Next annual spending cycle	End of fiscal evaluation year	OLS, LASSO, RF, and gradient boosting	LASSO models achieved test a R^2^ of 0.782 for hospital spending, with consumer expenditures contributing up to 33.6% of model fit.
44	Tan et al., 2024 [55]	Singapore	Forecast high healthcare utilization (inpatient bed days and ED visits) in patients with diabetes mellitus	Retrospective cohort study	111,004 patients	AUC-ROC, sensitivity, PPV, and PR-AUC	Baseline clinical assessment	Next 12 months (inpatient/ED visits)	1-year follow-up completion	LR, MARS, boosted trees, MLP, KNN, and BART	Random oversampling with boosted trees and MARS achieved robust discrimination (AUC-ROC > 0.80; sensitivity > 0.65).
45	Wang et al., 2025 [69]	United States	Predict prolonged emergency department wait times (≥30 min) among triage ESI-3 patient visits	Retrospective observational study	177,665 ED encounters	Accuracy, recall, precision, F1-score, AUC-ROC, FPR, and FNR	ED arrival/triage registration	≥30 min wait time	First physician/provider contact	CVLR, RF, XGBoost, ANN, and SVM	All models achieved comparable accuracy (0.74–0.75) and AUC-ROC values (0.78–0.82); crowding and arrival mode were the top predictors.
46	Wichmann et al., 2023 [38]	Brazil	Evaluate multicenter training data aggregation strategies for predicting in-hospital COVID-19 mortality	Multicenter cohort study	8477 patients (18 hospitals)	AUC-ROC, recall, and specificity	Hospital admission	In-hospital stay duration	Hospital discharge or death	XGBoost, LightGBM, and CatBoost	Local training using hospital-specific data achieved the highest performance in 61% of hospitals compared with regional pooling.
47	Xiaoqing et al., 2023 [52]	China	Predict outpatient waiting times across clinical departments in a tertiary children’s hospital	Retrospective cohort study	193,520 outpatient visits	R^2^ and MAE	Registration/check-in	Continuous wait time (minutes)	Call to physician consulting room	LR, KNN, GBDT, and RF	GBDT and RF outperformed LR, reducing the prediction MAE by up to 47.6% (MAE: 5.03 min in internal medicine).
48	Yang et al., 2025 [43]	China	Predict medical and daily care service demand among older adults using machine learning	Cross-sectional survey study	1291 elderly individuals	Accuracy, recall, precision, F1-score, and AUC-ROC	Cross-sectional assessment	Prospective care demand (6–12 months)	In-person follow-up assessment	RF, GBDT, and LightGBM (with SMOTE-Tomek Link)	LightGBM achieved the highest AUC-ROC values for medical (0.910) and daily care (0.906) demand; chronic disease count was the top predictor.
49	Zalikha et al., 2023 [70]	United States	Compare ML algorithms and evaluate patient-specific vs. situational variables in predicting total knee arthroplasty outcomes	Retrospective cohort study	305,577 primary TKA discharges	AUC-ROC accuracy	Preoperative/discharge	In-hospital and 30-day complications	30 days post-arthroplasty	RF, NN, XGBoost, LSVM, CHAID, decision list, LR, and Bayes Net	Linear SVM achieved superior responsiveness and accuracy; patient-specific variables consistently outperformed situational variables.
